# Chitinolytic enzymes of the rumen ciliate *Eudiplodinium maggii*

**DOI:** 10.1007/s12223-012-0133-6

**Published:** 2012-04-13

**Authors:** R. Miltko, G. Belzecki, T. Michalowski

**Affiliations:** The Kielanowski Institute of Animal Physiology and Nutrition Polish Academy of Sciences, Instytucka 3, 05-110 Jabłonna near Warsaw, Poland

## Abstract

The ability of rumen ciliates to digest chitin is clearly recognized. We investigated the chitinolytic system of the rumen ciliate *Eudiplodinium maggii*. The ciliates were grown in a selectively faunated sheep. They were isolated from the rumen and purified by sedimentation. A crude enzyme preparation was prepared following incubation of ciliates with antibiotics. This was done in order to reduce their contamination with intracellular bacteria. The activity of particular enzymes was examined by quantification of the products released from specific substrates. It was stated that the optimum conditions for the detected activities varied between 4.5 and 5.5 pH, and 45 and 55 °C. β-*N*-Acetylglucosaminidase was found as an enzyme of the highest activity (4.2 μmol/l released product per mg protein per h). The activities of endochitinase and exochitinase were almost two times lower than that of β-*N*-acetylglucosaminidase. Zymographic studies revealed the presence of two endochitinases, two exochitinases and two β-*N*-acetylglucosaminidases in the examined preparation.

## Introduction

Rumen ophryoscolecid protozoa engulf readily the fungal zoospores which are rich in chitin (Williams and Coleman 1997; Lee et al. 2001). We already found that the ciliates *Eudiplodinium maggii* digest and ferment chitin (Miltko et al. 2010). However, no information is available on chitinolytic enzymes of this species of rumen protozoa. The objective of this study was to identify and characterize chitinolytic enzymes of this species of ciliates.

## Materials and methods

The ciliates *E. maggii* were identified after Dogiel (1927). They were isolated from the natural rumen fauna of sheep. The ciliates were cultured under in vitro conditions according to Michalowski et al. (1991) and were then inoculated into the rumen of ciliates-free sheep (Michalowski et al. 1999). The ciliates living in the rumen of monofaunated sheep (see above) were used to perform the enzymic experiments. The samples of the fluid (about 1 l) were withdrawn from the rumen and the protozoa were isolated and purified by sedimentation (Michalowski 1990). The purified ciliates were incubated overnight with a mixture of antibiotics (chloramphenicol, streptomycin and ampicillin) each of which was supplied at the final concentration of 50 μg/ml. The antibiotics were used in order to restrict the intracellular bacteria. After incubation, the ciliates were washed three times with warm (40 °C) caudatum type salt (Coleman et al. 1972). Finally, they were concentrated by the sedimentation method and stored at −80 °C. To obtain the enzyme preparation the frozen ciliates were thawed and homogenized in a glass homogenizer equipped with a Teflon pestle. The resulting homogenate was centrifuged (22,000×*g*, 30 min, 4 °C) and the supernatant was collected and used as a crude enzyme preparation. The activity of endochitinase was determined by quantification of reducing sugars released from carboxymethylchitin (Wirth and Wolf 1990) following its incubation with crude enzyme preparation. Reaction mixture consisted of 0.4 ml substrate, 0.4 ml enzyme preparation and 0.2 ml 0.1 mol/l citrate–phosphate buffer. The mixture was incubated for 1 h at 40 °C and the released products were measured spectrophotometrically according to Miller et al. (1960). The exochitinase and β-*N*-acetylglucosaminidase activities were determined by measurements of nitrophenyl released by crude enzyme preparation from 4-nitrophenyl-*N,N-*diacetyl-d-chitobioside and 4-nitrophenyl-β-*N*-acetylglucosaminide, respectively. Reaction mixture consisted of 100 μl solution of 1 μmol/l substrate, 50 μl enzyme preparation and 150 μl 0.1 mol/l citrate–phosphate buffer. It was incubated for 1 h at 40 °C and the released product was quantified according to Yem and Wu (1976). Native polyacrylamide gel electrophoresis (PAGE) of crude enzyme preparation in combination with zymography technique was applied to identify the chitin degrading enzymes (Wirth and Wolf 1990). Carboxymethylchitin, 4-methylumbelliferyl-β-d-*N*,*N*-diacetylchitobioside and 4-methylumbelliferyl-*N*-acetyl-β-d-glucosaminide were added as the specific substrates to the separating gels to identify endochitinase, exochitinase and β-*N*-acetylglucosaminidase, respectively.

## Results and discussion

We showed that *E. maggii* ciliates possess endochitinase, exochitinase and β-*N*-acetylglucosaminidase which were responsible for the breakdown of chitin. This finding supports the earlier results concerning of chitinolytic properties of *Diploplastron affine* (Belzecki et al. 2008). They showed also that the most active was β-*N*-acetylglucosaminidase. It was 12 times more active than the similar enzyme which was found in *E. maggii* by Williams et al. (1986). The endochitinase and exochitinase were similar in their activities (*p* > 0.05) which were about two times lower than β-*N*-acetylglucosaminidase (Table 1).

**Table 1:** Characterization of chitinolytic enzymes of *Eudiplodinium maggii* ^a^

Chitinolytic activity	pH optimum	Temperature optimum (°C)	Degradation rate (μmol/l released product per mg protein per h)
Endochitinase	5.5	45–55	1.7
Exochitinase	4.5–5.0	45	2.0
β-*N*-Acetylglucosaminidase	4.5	55	4.2*

^a^Mean values (*n* = 3)

*p < 0.05

**Fig. 1:**
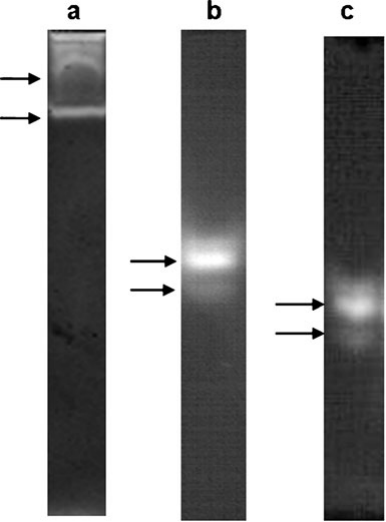
Chitinolytic enzymes of the rumen ciliate *Eudiplodinium maggii* identified by the zymogram technique; protozaol proteins were separated by native PAGE. **a** Endochitinases, **b** Exochitinases, **c** β-*N*-acetylglucosaminidases; *arrows* bands exhibiting chitinolytis activity

In general, six protein bands exhibited the ability to degrade chitin or its derivatives (Fig. 1). Two of them were endochitinases, two were exochitinases, and two were β-*N*-acetylglucosaminidases.
